# Multipolar passive cloaking by nonradiating anapole excitation

**DOI:** 10.1038/s41598-018-30935-3

**Published:** 2018-08-21

**Authors:** Anar K. Ospanova, Giuseppe Labate, Ladislau Matekovits, Alexey A. Basharin

**Affiliations:** 1 National University of Science and Technology (MISiS), Department of Theoretical Physics and Quantum Technologies, 119049 Moscow, Russia; 2 National University of Science and Technology (MISiS), The Laboratory of Superconducting metamaterials, 119049 Moscow, Russia; 3 Politecnico di Torino, Department of Electronic and Telecommunications, 10129 Torino, Italy

## Abstract

In this paper, we demonstrate the relation between cloaking effect and its nonradiating state by considering the destructive multipolar interaction between near-field scattering by bare object and surrounding coating located in its proximity. This cloaking effect is underpinned by anapole mode excitation and it occurs as destructive interference between the electric dipole moment, generated by a bare object (here a central metallic scatterer) and the toroidal moment, formed inside the cloak (a surrounding cluster of dielectric cylinders). Numerical results show how a cloaking effect based on the formation of the anapole mode can lead to an overall nonradiating metamolecule with all-dielectric materials in the coating region.

## Introduction

Pioneering works on invisible bodies and transparent objects have strongly contributed to the concept of electromagnetic invisibility^1–4^, that has passed from science fiction to science in the last decade with the excitation of nonradiating states^5–8^ and the introduction of cloaking devices^9–14^. Other techniques such as (i) camouflaging, popular for military purposes, have been explored by shaping the object’s contour with different designs and (ii) acoustic/thermal footprint reduction approaches^15,16^, thus with the consequent redirection or the absorption of the impinging vector/scalar field. Another invisibility technique is the transparency phenomenon, inspired by what happens at the quantum level with the incident wave that excites complex level transitions and, at the same time, interferes destructively with them at resonance. This occurrence leads to sharp and narrow transparency peaks in the absorption spectrum: similarly, analogous interferences can be implemented in classic systems and gives rise to full transparency according to classical electrodynamics. Depending on the complexity of the system, this effect can be explained as a particular case of Fano-resonance, a classical analogues of electromagnetically induced transparency (EIT) like metamaterial induced transparency (MIT), plasmon-induced transparency (PIT) and others^17–20^.

The interest in the nonradiating feature of electromagnetic invisibility has been developed starting from a set of theorems by Devaney and Wolf^21^, highlighting the mathematical properties of nonradiating currents. On the other hand, nowadays, the most exciting invisibility technique is related to cloaking devices, where the interaction of the impinging wave with a coating, surrounding the object to be hidden, leads to the generation of subset of nonradiating currents. The development of cloaking devices has advanced with the improvement of metamaterials, exploiting the fact that subwavelength media exhibit unnatural properties due to the geometry and spatial arrangement of their unit cells, hereafter called *metamolecules*^22–28^. These usually composite materials could manifest negative refraction index, strong field localization and other exotic properties that make them strategic in many research areas^22,29^.

Several techniques have been proposed to realize cloaking devices: the most popular of them are Transformation Optics (TO)^10,11,30^, Scattering Cancellation, subdivided in Plasmonic Cloaking (PC)^9,31^ and Mantle Cloaking (MC)^12,13,32^, and other approaches have been pursued in the literature^33,34^. TO is based on a spatial transformation of a certain region close to the near-field of the object itself, bending the path of the electromagnetic waves with bulk metamaterial coatings^17,33,35^. If properly manipulated, the effect is to maintain free-propagating characteristics all around the region of interest by rerouting the energy flow in such a way that electromagnetic waves do not interact with the hidden object at all. On the other hand, PC exploits the coating-object interaction at plasmon excitation frequencies of the coating in order to lead to a dramatic reduction of the scattering cross section of the cloaked object^9^ with respect to the bare counterpart. This scattering cancellation approach belongs also to the MC technique, which differs from the PC and TO by replacing the volumetric metamaterial coating with a patterned ultrathin “mantle cloak”. This method drastically suppresses the scattering from planar (1D), cylindrical (2D) or spherical (3D) objects and it can be realized by means of metasurfaces or frequency-selective surfaces (FSS)^12,13^. Nowadays, a growing number of research efforts has been performed towards new types of improved covers with better transmission and/or reflection properties, miniaturized sizes or increased operational bandwidth^36^, just to mention some of the most challenging aspects of this contemporary research area.

In this paper, we demonstrate for the first time the relation between nonradiating states named as anapoles^6–8,37^ and the cloaking effect based on the multipolar interactions between the object to be hidden and the coating around it. Even if developed as separate research areas, the nonradiating and cloaking literatures have been only recently put together in order to derive compact solutions for the invisibility problem^38,39^. This is because it was supposed that separate phenomena occur when the excitation comes from the inside of the structure (nonradiating problem) or from an external incident wave (cloaking problem). In this work, we show how the nonradiating anapole state that is excited by an internal dipole antenna in^8^ can be induced as well from an external electromagnetic wave impinging on a complete passive structure. In contrast with the aforementioned cloaking techniques, the nonradiating feature of the induced currents that give rise to a destructive interference due to the interplay of properly excited multipoles in both the object and coating region explains electromagnetic invisibility. By considering the excitation in the near-field between external primary waves and induced secondary sources, the overall structure becomes nonradiating for an internal observer and, at the same time, it appears as cloaked for an external observer: in the following section, we present and describe the theory behind this technique, called as *multipolar cloaking*.

## Results

### Multipolar cloaking technique

The “multipolar cloaking” technique is here introduced by considering a simple yet effective model of general type. Any subwavelength object is characterized by an electric or magnetic dipole moment that well approximates its scattering at the first order of a multipole expansion. In order to suppress the electromagnetic scattered field generated by such an object, one should make it interacting with a medium that exhibits the *same kind* of multipole moment. In particular, toroidal dipole moment **T** has the same far-field properties as the electric dipole moment **P** and this enables destructive interference between them in the far-field, providing that **P** = *ik***T**^5^, where *i* denotes the imaginary unit and *k* is the propagation wave vector.

Our purpose in the present paper is to find a surrounding cloak with purely toroidal moment **T** that, together with the dipole moment **P** of the bare scattering object, creates a nonradiating state, which is by definition an anapole mode^5–8^. In the ideal case, this nonradiating state achieves complete scattering cancellation of electromagnetic fields in the far-field region and the destructive interference between electric and toroidal dipole moments can be formulated as “multipolar cloaking”.

The toroidal dipole moment is the third order term in the multipole decomposition of the near-field with respect to the usually considered expansion (it comes after electromagnetic dipoles and quadrupoles) and, together with electric and magnetic moments, with its toroidal topology^40,41^ it contributes to the near-field interaction of electromagnetic field. This configuration supports poloidal currents, existing along the meridians of the surface of a torus that in turn provides loops of magnetic vortices along the torus: this arrangement of poloidal currents and magnetic vortices generates a dynamic toroidal response, directed along the symmetry axis of the torus. Despite the fact that toroidal and electric moments are defined by different current configurations, their angular moments and radiation patterns are identical in the far-field zone. If both are present within a structure, destructive interference between electric and toroidal moments can occur and, by definition, this sustains an anapole mode: in the ideal case, it corresponds to a strong field localization confined around a point source accompanied by suppressed radiation outside. Experimental confirmations of strong toroidal response in metal and all-dielectric metamaterials are given in^42^ and^43^, respectively. For the first time, the existence of an anapole mode in metamaterials has been studied in^5^ for a dumbbell-shaped metaparticles and for a single all-dielectric disk^44^. In addition, electrodynamics of anapole mode excitation has been described by Afanasiev and Dubovik, Radescu and Vaman^45,46^ with promising applications for such electromagnetic mode in nanophotonics due to strong field localization, lasing and spaser regimes^44,47–56^.

In order to implement our model with the presence of both electric and toroidal moments, we choose as a bare object, for a very large dipolar response, a perfect electric conductor (PEC) cylinder whereas, for a strong toroidal response, we choose as a cloak, a high-index all-dielectric cluster^7,8,57^. When combining object and cloak together, a destructive interference between electric dipole contribution (coming from the bare object) and the toroidal contribution (coming from the cloak) is expected, guaranteeing invisibility of the structure and ideal cloaking conditions in a given frequency range.

### The structure of the system

In particular, the cloaking device considered here is a metamolecule, made up of four subwavelength high-index dielectric cylinders, and a bare PEC cylinder in the center of the structure.

The properly designed geometry allows creating a strong toroidal response in the four lateral dielectric cylinders and a strong electric dipole response by the central scatterer, leading to the appearance of the anapole mode. The exploitation of the dielectric particles in the cloak region is justified for their low dissipative losses and, moreover, the structure itself is free from complex geometrical patterns thanks to its simplicity of fabrication.

**Figure 1:**
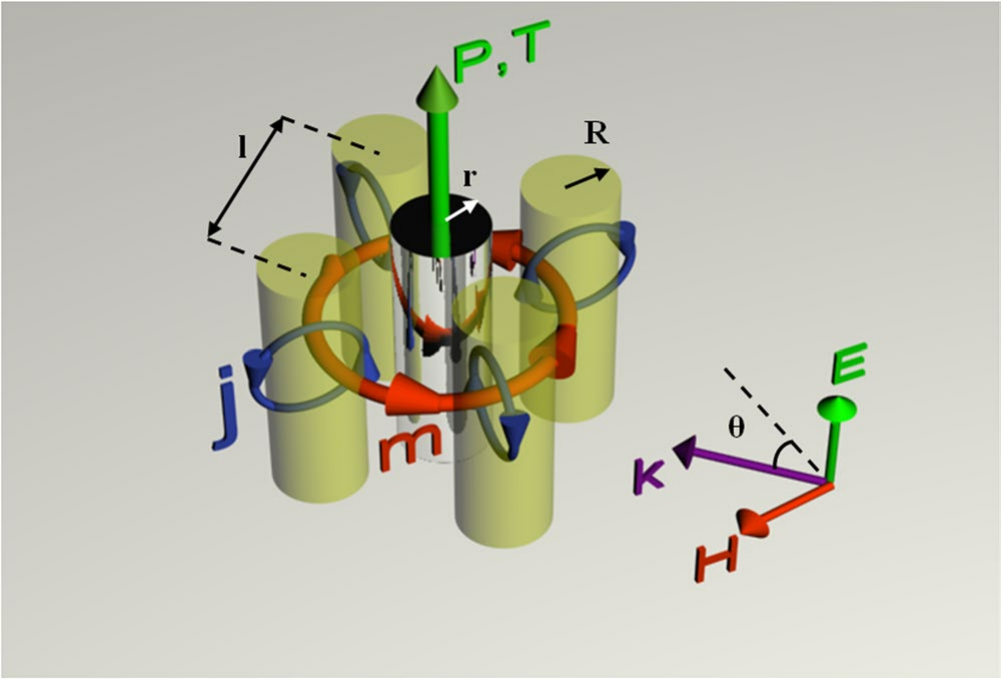
Illustration of the multipolar cloaking metamolecule, consisting of four dielectric and one central PEC cylinders. The electric component of the linearly polarized incident wave is parallel to the cylinders’ axis. Here *R* = 5 μm is the radius of dielectric cylinders, *r* = 2.5 μm is the radius of the PEC scatterer, *l* = 12 μm is the center-to-center distance between cylinders and θ denotes the angle of incidence with respect to one of the symmetry axis of the metamolecule. **P** and **T** stands for electric and toroidal moments, respectively; **j** denotes displacement currents and **m** denotes magnetic moment.

The multipolar cloaking metamolecule is schematically illustrated in Fig. 1. The four high-index dielectric cylinders have the same radius *R* = 5 μm and positioned in a square-shape geometry with center-to-center distance *l* = 12 μm. The radius of the central metallic cylindrical scatterer is *r* = 2.5 μm. In order to simplify the numerical simulations, we consider the height *h* of each cylinder to be indefinitely elongated and the whole metamolecule placed in vacuum. In particular, the electromagnetic response of the metamolecule is governed by the displacement currents induced in each dielectric cylinder and central scatterer by an impinging electromagnetic wave, with the E-field directed along the cylinder’s axis (TM polarization). The relative permittivity of the four all-dielectric cylinders is *ε*_*r*_ = 41.4 which is very close to the dielectric constant of LiTaO_3_ in the low THz regime^58,59^.

Depending on the geometrical parameters of the metamolecule (radius *r* and center-to-center distance *l* between cylinders) and permittivity of the considered materials (as well as polarization and angle of incidence for the incoming wave), the electromagnetic scattering produced by the displacement currents in the cloak (and surface currents on the object) could be controlled and, in particular, it becomes resonant for some configuration. Commercial Maxwell’s equation solver CST Microwave Studio has been used to study the electromagnetic response in frequency of the proposed configuration, aiming to understand cloaking mechanism, that can be described as follows:

1. The electromagnetic response of the cloaking metamolecule is underpinned by the loop-shaped displacement current **j**, induced within each dielectric cylinder by the impinging parallel-polarized wave (Fig. 1). According to Mie theory, each displacement current loop **j** causes magnetic mode **m** within each cylinder. The proper arrangement of cylinders leads to a head-to-tailed loop of magnetic fields, resembling magnetic vortex inside the simplified torus. This dipolar configuration mimics electromagnetic properties of a torus and produces a toroidal moment **T** directed along the center of metamolecule and parallel to its axis.
2. The presence of the central PEC cylinder is responsible for the surface currents excited at its circular boundary or, from a multipolar expansion point of view, for the electric moment **P** of the metamolecule.

For this reason, it is possible for an externally incident electromagnetic wave to create both toroidal response **T** – in the cloak with the dielectric cylinders - as well as electric dipolar response **P** – in the bare conductive cylinder. The resonant response of the cloaking metamolecule, accompanied by a phase shift between electric **P** and toroidal **T** dipole moments, corresponds to anapole mode excitation if the **P** = *ik***T** relation holds. In such condition, an external observer cannot detect the metamolecule, since the incoming wave goes through unperturbed due to the nonradiating feature of the above described anapole mode, underlying the reason for multipolar cloaking effect. It is expected that the described mechanism can be used as a solution for the problem of nano-objects cloaking, since most nanoparticles and molecules are characterized by strong electric dipole moments and they can be masked by all-dielectric surrounding nanoparticles with strong toroidal moment.

### Scattering properties and field distribution of multipole cloaking metamolecule

Efficient omnidirectional, i.e., isotropic, cloaking requires effective scattering cancellation in all directions. The scattering properties of multipolar cloaking device has been estimated by calculating the Radar Cross Section (RCS), defined in the far-field, for the following three cases: single scatterer (PEC cylinder), the dielectric cloak (cluster of four all-dielectric cylinders) as well as the combination of PEC cylinder within all-dielectric objects, forming the metamolecule or cloaking device.

**Figure 2:**
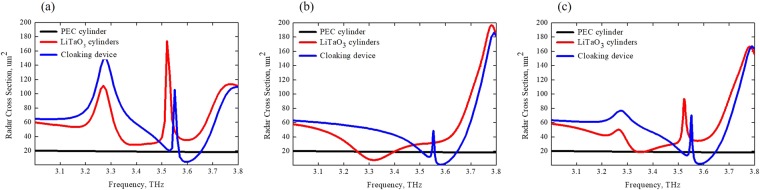
Radar cross section (RCS) for bare PEC object (black curve), all-dielectric cover (red curve) and cloaking metamolecule (blue curve) for parallel polarization of incident wave at θ = 0° (**a**), at θ = 45° (**b**) and at θ = 30° (**c**) incidence angles.

RCS calculation for single PEC scatterer (black curve), for all-dielectric cluster (red curve) as well as for cloaking device (blue curve) are presented in Fig. 2 for three different orientations of the incident wave, namely *θ* = 0°, *θ* = 45°, *θ* = 30°. The radius of the central scatterer is much less than the one for the dielectric cylinders in terms of working wavelength and provides RCS level of 20 μm^2^ for *r* = 2.5 μm. For the case *θ* = 0° (Fig. 2(a)), the all-dielectric cluster (red curve) has two scattering minima at 3.38 THz (28.6844 μm^2^) and 3.58 THz (35.1515 μm^2^), corresponding to sufficiently large scattering losses. Nevertheless, the RCS for cloaking device at the same frequency (3.58 THz) is suppressed down to 4.46 μm^2^ due to the coupling between all-dielectric cluster and PEC cylinder (blue curve): it is about 8 times smaller than the one for all-dielectric cluster and 5 times smaller than the one for the bare PEC cylinder. Figure 2(b,c) show results for cloaking device at *θ* = 45° and *θ* = 30° incidence angles at f = 3.58 THz accompanied by suppressed scattering of 0.98 μm^2^ and 0.2 μm^2^, respectively. Thus, the results reported in these figures confirm that the insertion of a strong PEC scatterer with toroidal all-dielectric coating results in dramatically suppressed scattering for both of them.

**Figure 3:**
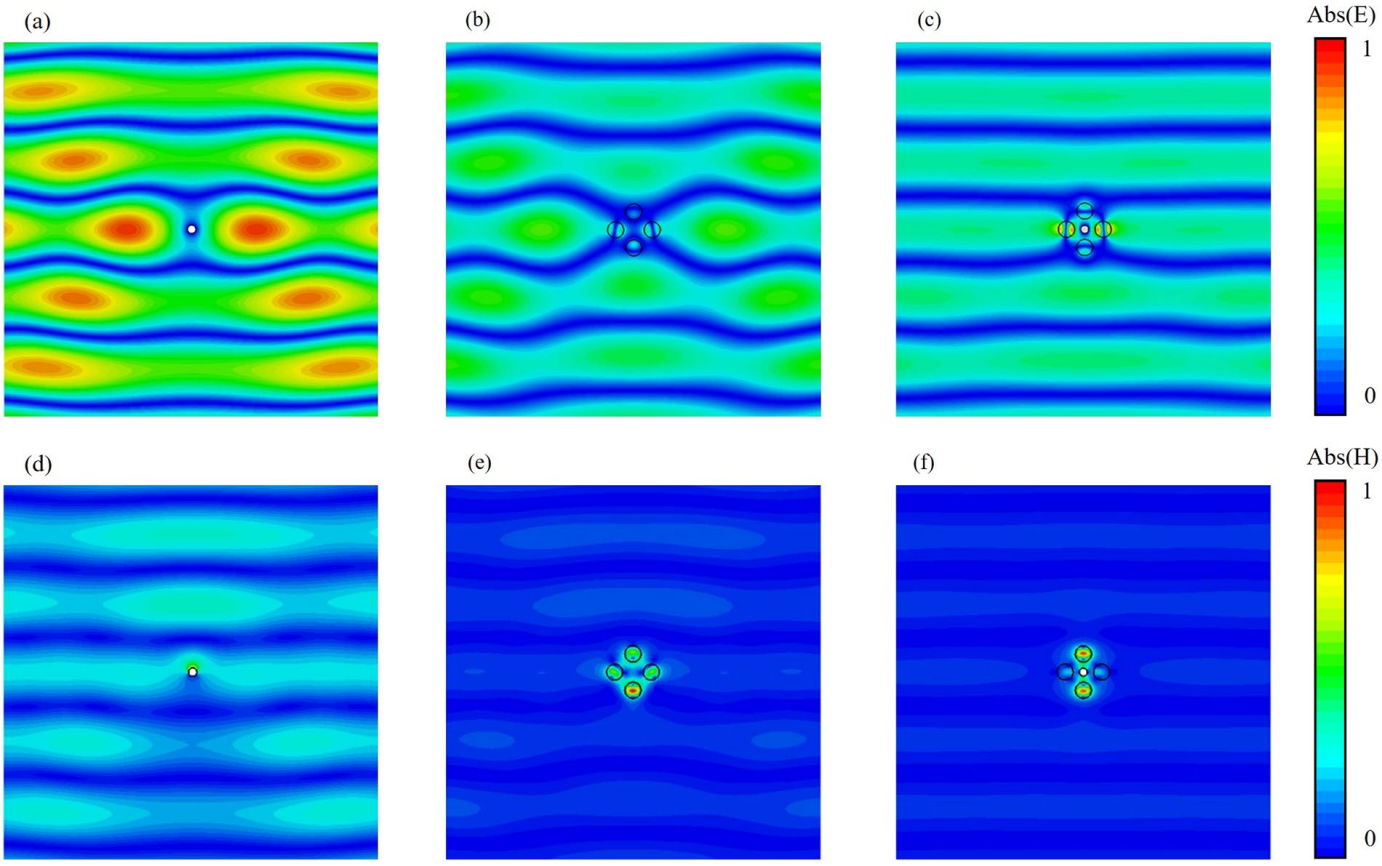
Field maps of the y-component (along cylinders’ axis) of electric field intensity and the absolute value of magnetic field (across cylinders’ axis) intensity of bare PEC object (**a**) and (**d**), all-dielectric cover (**b**) and (**e**) and cloaking metamolecule (**c**) and (**f**) for θ = 45° incident wave at resonant frequency f = 3.58 THz.

Furthermore, the field distribution at the frequency of minimum scattering for all the three cases of arrangement have been studied: single PEC scatterer, all-dielectric cluster and cloaking metamolecule. Figure 3 shows the field maps for the y-component (along cylinders’ axis) of electric field intensity and absolute value of magnetic field intensity (orthogonal to the cylinders’ axis) of single PEC scatterer (Fig. 3(a,d)), all-dielectric cluster (Fig. 3(b,e)) and cloaking device (Fig. 3(c,f)) for θ = 45° incident wave at the resonant frequency f = 3.58 THz.

The electric and magnetic fields of PEC cylinder (Fig. 3(a,d)) are expected to be characterized by a strong distortion of the wavefront. Quite similar results are observed for electric and magnetic field distributions of all-dielectric cluster (Fig. 3(b,e)). The reason is due to the fact that electric and magnetic fields distribution of bare PEC cylinder and all-dielectric cluster represent dipole and toroidal response, respectively. On the other hand, from the overall cloaking device, one can see almost unperturbed electric and magnetic field distributions of the metamolecule (Fig. 3(c,f)). This is due to the destructive interference between near-fields of PEC scatterer and all-dielectric cluster that leads to undistorted wave propagation through the cloaking metamolecule that sustains an anapole mode. Supplementary Figs S1 and S2 provides the field maps of electric and of magnetic field intensities for cloaking device at θ = 30° and 0° incidence angle.

This is to demonstrate that our assumption of inducing, with an external electromagnetic wave, a nonradiating state between a central electric scatterer and a collection of scattering objects around is correct. In this respect, this geometrical configuration of a central object and other scattering “satellites” around it is very convenient as highlighted in^31^, due to the fact that the cloaked object is not in direct contact with the coating and it is not fully covering the structure, but a discrete finite number of objects can be used. However, the cloaking mechanism demonstrated in^31^ is of PC type and, since the destructive interference is considered to be a dipole-dipole interaction between the object and its cloak, plasmonic materials with negative permittivity have to be used in the scattering satellites in order to achieve an antiphase contribution. In our suggestion, the cloaking mechanism is due to a destructive multipolar interaction for a central electric scatterer, acting as a bare object with dipole response: here an all-dielectric coating with positive permittivity is employed since the destructive interference is considered to be a electric-toroidal interaction.

**Figure 4:**
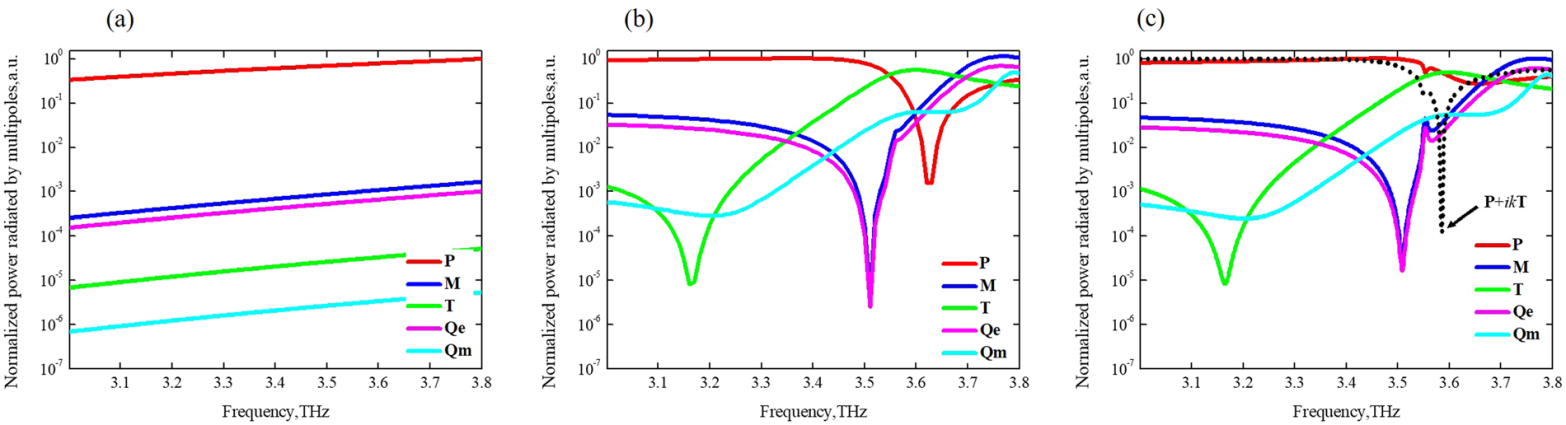
Normalized power of near-field distribution of PEC-scatterer (**a**), dielectric coating (**b**) and cloaking metamolecule (**c**) up to fifth terms of multipoles for θ = 45° incident wave direction.

In confirmation of our assumption, we provide multipole decomposition of near-fields excited by the external incident wave in the cloaking metamolecule in terms of the five strongest multipoles: electric dipole **P**, magnetic dipole **M**, toroidal dipole **T**, electric quadrupole **Qe** and magnetic quadrupole **Qm** moments, respectively. Figure 4 and Supplementary Fig. S3 provide multipolar decomposition up to the fifth terms of multipoles scattered by cloaking device at the frequency range 3–3.8 THz. Single PEC cylinder, as expected, shows a strong electric dipolar **P** response exceeding other multipoles more than 1000 times (Fig. 4a). Figure 4(b) shows multipole decomposition of all-dielectric cluster with dominating toroidal dipole **T** around 3.58 THz at θ = 45° angle of incidence. Finally, it is possible to observe a well-pronounced anapole mode establishment for multipolar cloaking device at 3.58 THz (Fig. 4(c)). Electric dipole moment **P** dominates at low frequencies and falls dramatically at 3.63 THz, while toroidal dipole **T** gradually increases from 3.167 THz and remains dominant from 3.551 THz to 3.671 THz. At the same time, magnetic dipole **M** and electric quadrupole **Qe** moments grow rapidly from 3.5 THz up to 3.727 THz, when they become almost dominant and the magnetic quadrupole **Qm** keeps growing in the whole frequency range. Furthermore, the total interference of electric dipole **P** and toroidal dipole **T** moments is shown in the form **P** + *ik***T** (amplitude of which is represented by the black dashed curve) and this confirms strong scattering suppression at f = 3.58 THz due to anapole mode establishment.

We should stress that ideal multipolar anapole cloaking is determined by interference of electric and toroidal moments in the absence of other kind of excitations, i.e. higher order multipoles. This kind of definition is convenient for analysis of multipolar interaction while non-appropriate for real cases. However, in real multipolar cloaking, interference of electric and toroidal moments is accompanied by unavoidable perturbations, such as strong magnetic response and higher order multipole excitations. Our simulations, accompanied by rather high values of magnetic dipole **M** as well as of electric **Qe** and of magnetic **Qm** quadrupoles, are justified and acceptable in real multipole cloaking approach, that radiates and induces extra, even if low, far-field scattering.

**Figure 5:**
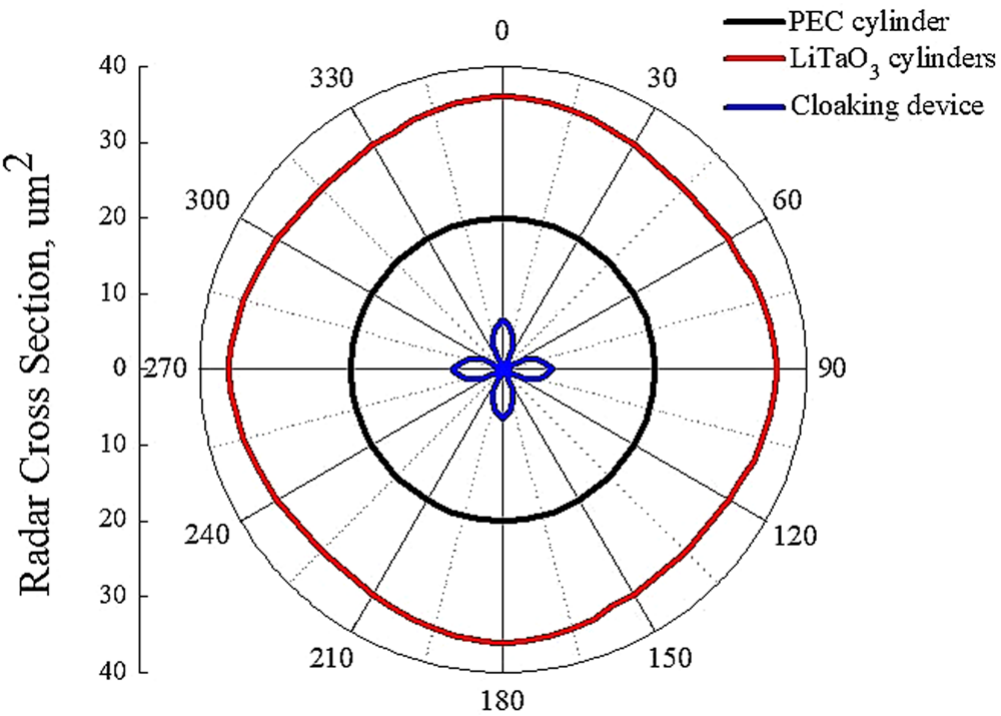
RCS pattern of PEC scatterer (black curve), all-dielectric coating (red curve) and multipolar cloaking device (blue curve) at minimum scattering frequency value of f = 3.58 THz.

Furthermore, we provide RCS pattern for PEC cylinder (black curve), all-dielectric cluster (red curve) and multipolar cloaking device (blue curve) in Fig. 5. The map of the far-field distribution is reported for minimum scattering value of the RCS at f = 3.58 THz for the cloaking device (see Fig. 2). Interestingly, far-field distribution pattern for all-dielectric cluster (red curve), accompanied by toroidal dipole mode, strongly resembles the pattern of electric dipole scattering (black curve), exceeding in radiation rate up to 2 times. This confirms the radiating nature of the toroidal dipole, which in far-field is indistinguishable from that of the electric dipole. On the other hand, the cloaked metamolecule (blue curve) possesses much less scattering signature. Depending on the incidence angle, the RCS varies from 0.98 μm^2^ at *θ* = 45° up to 4.46 μm^2^ for *θ* = 0° angle of incidence. This global RCS pattern can be explained by looking at the multipole decomposition. Indeed, anapole mode at f = 3.58 THz suppresses radiation of electric type, i.e., that described in terms of **P**. However, the residual higher order multipoles, such as magnetic quadrupole **Qm**, magnetic dipole **M** and electric quadrupole **Qe**, are responsible for the four lobes that appears in the RCS diagram for the cloaking metamolecule (blue curve on Fig. 5).

## Discussion

Our idea of multipolar cloaking with subwavelength objects based on the interaction of excited multipoles by the impinging wave can be assumed as a methodology for the design of all- dielectric coatings. Based on this approach we put an idea to compensate the scattering from hiding objects by counter-propagating the scattering from a coating with toroidal response. Such interaction of the impinging wave with the cloaking metamolecule represents a promising future for development of devices in many fields. From the application ranges point of view, one should mention that nondestructive observations at the nanoscale systems for subwavelength cloaking techniques are long awaited. For instance, invasive insertion of electric probes leads to strong near-field redistribution. However, the insertion of subwavelength electric probes, that appear as “cloaked”, leads to unperturbed near-field distribution of the medium to be studied, i.e., giving rise to the so called noninvasive probe. This low-level perturbation instrument is highly demanding in extended research activity in medicine and biology, where nanoscopic imaging of nanoparticles and molecules of different nature is required. Some of the measurements are performed in the near-field of the nanoparticle and demand low-level perturbation, also seen as noise levels for the sensing instruments with limited accuracy of measurements. The solution could be found in the form of “cloaking” the sensor^60^, which does not disturb near-field distribution. One can exploit this in biology and medicine for minimizing the interference on near-field distribution. Multipolar cloaking based sensing devices can advance the diagnostics of living organisms without disturbance other life systems. The spread of cancer causes the need for destruction of cancer cells with specific radiation without harming living cells. Since multipolar cloaking has narrow bandwidth, it can be tuned to preferred mode operation and it is expected to exceed many probes in terms of sensitivity. Namely, experiments in plasma physics suffer from probe invasion, which strongly interacts with near-field potential of plasma such that it falsifies results of measurement or strongly reduces accuracy. Thus, the proposed cloaking can be used as well-tuned non-invasive probe both for nanophotonics and nanomedicine as a nondestructive sensing devices^61,62^.

On the other hand, such cloaking regime can be used to switch on/off radiating/nonradiating states, i.e., realizing strongly high-quality sensing device. When an electric scatterer is surrounded by a proper coating layer, at certain frequencies it acts as an open resonator with high Q-factor, while not interacting with the surrounding media: the proposed technique could be involved also for this possible application^29^.

## Conclusion

In this work, we have proposed the idea of multipolar cloaking of electric scatterer by means of anapole mode excitation. We have shown that toroidal moment of all-positive dielectric cluster falls into destructive interaction with the electric moment of PEC scatterer and both of them become cloaked, i.e., exhibiting minimum scattering value. Regarding to multipole decomposition technique, this kind of cloaking, indeed, is based on anapole mode excitation, which corresponds to nonradiating state with strong field localization within cloaking metamolecule and strong scattering suppression outside its domain of definition. Such device could be demanding in a number of industrial fields, as noninvasive sensor in biology and medicine, strong field-localizing electric devices, nonradiating source and others.

## Methods

### Simulations

The electromagnetic properties of the metamolecule made up of metal and dielectric have been computed with the aid of a commercial Maxwell’s equation solver CST Microwave Studio using the standard transient modeling approach. The simulations provide values of RCS, giving data on scattering properties of metamolecule. The simulations also provide the data of electrical currents densities induced in metallic parts of the source and displacement currents in dielectric cylinders, which are used to calculate the powers radiated by conventional single multipoles, including those of toroidal dipoles.

## Electronic supplementary material

Supplementary Information
