# Supplementary material for: Multipolar passive cloaking by nonradiating anapole excitation
Source: Sci Rep. 2018 Aug 21;8:12514. doi: 10.1038/s41598-018-30935-3 (PMC6104082; doi:10.1038/s41598-018-30935-3)
Supplement: Supplementary file 1 — Supplementary Information [file 41598_2018_30935_MOESM1_ESM.docx]

Supplementary Information

**Multipolar passive cloaking by nonradiating anapole excitation**

Anar K. Ospanova^1, 2, 3^, Giuseppe Labate^3^, Ladislau Matekovits^3^, Alexey A. Basharin^1, 2, 3, *^

^1^ National University of Science and Technology (MISiS), Department of Theoretical Physics and Quantum Technologies, 119049, Moscow, Russia

^2^ National University of Science and Technology (MISiS), The Laboratory of Superconducting metamaterials, 119049, Moscow, Russia

^3^ Politecnico di Torino, Department of Electronic and Telecommunications, 10129, Torino, Italy

Correspondence and requests for materials should be addressed to A.A.B. (alexey.basharin@gmail.com)

Here, we provide information for cloaking device for the different angles of incident wave.

We represent on Fig. S1 the field maps of electric and magnetic fields distribution for all-dielectric cluster (Fig. S1 (a) and (c)) and cloaking device (Fig. S1 (b) and (d)) for θ=0^0^ angle of incident wave at resonant frequency f=3.58 THz. We show that incident wave front propagates freely through the multipole cloaking metamolecule (Fig. S1 (b) and (d)). Thus, the cloaking device behaves as nonscattering object with strong electromagnetic field concentration within cylinders. However, we note that all-dielectric cluster behaves as effective scatterer distorting wave front of impinging wave (Fig S1 (a) and (c)).


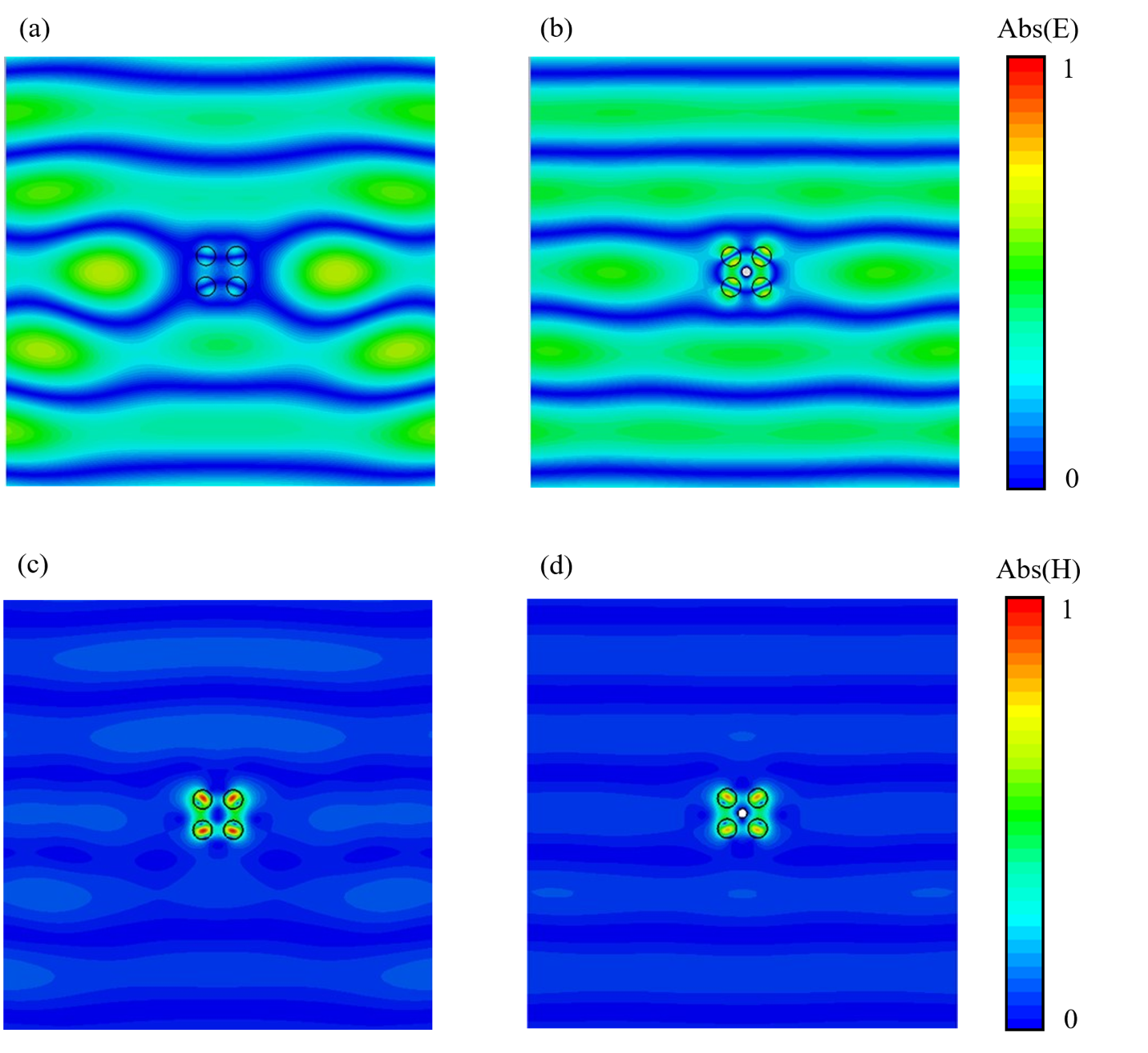


Fig S1. Field maps of the y-component of electric field intensities and of the absolute value of magnetic field intensities of all-dielectric cluster ((a) and (c)) and cloaking metamolecule ((b) and (d)) for θ=0^0^ incident wave at f=3.58 THz.

Almost identical fields distribution can be observed in Fig. S2 for all-dielectric cluster and cloaking device at θ =30^0^ incidence angle. Moreover, electric field on Fig. S2 (a) and magnetic field on Fig. S2 (c) show strong perturbation of electromagnetic wave around of all-dielectric cluster with toroidal mode excitation in metamolecule at f=3.58 THz. In case of cloaking device, incident wave propagates through cloaking metamolecule with negligible perturbation of electric and magnetic component of incident wave.


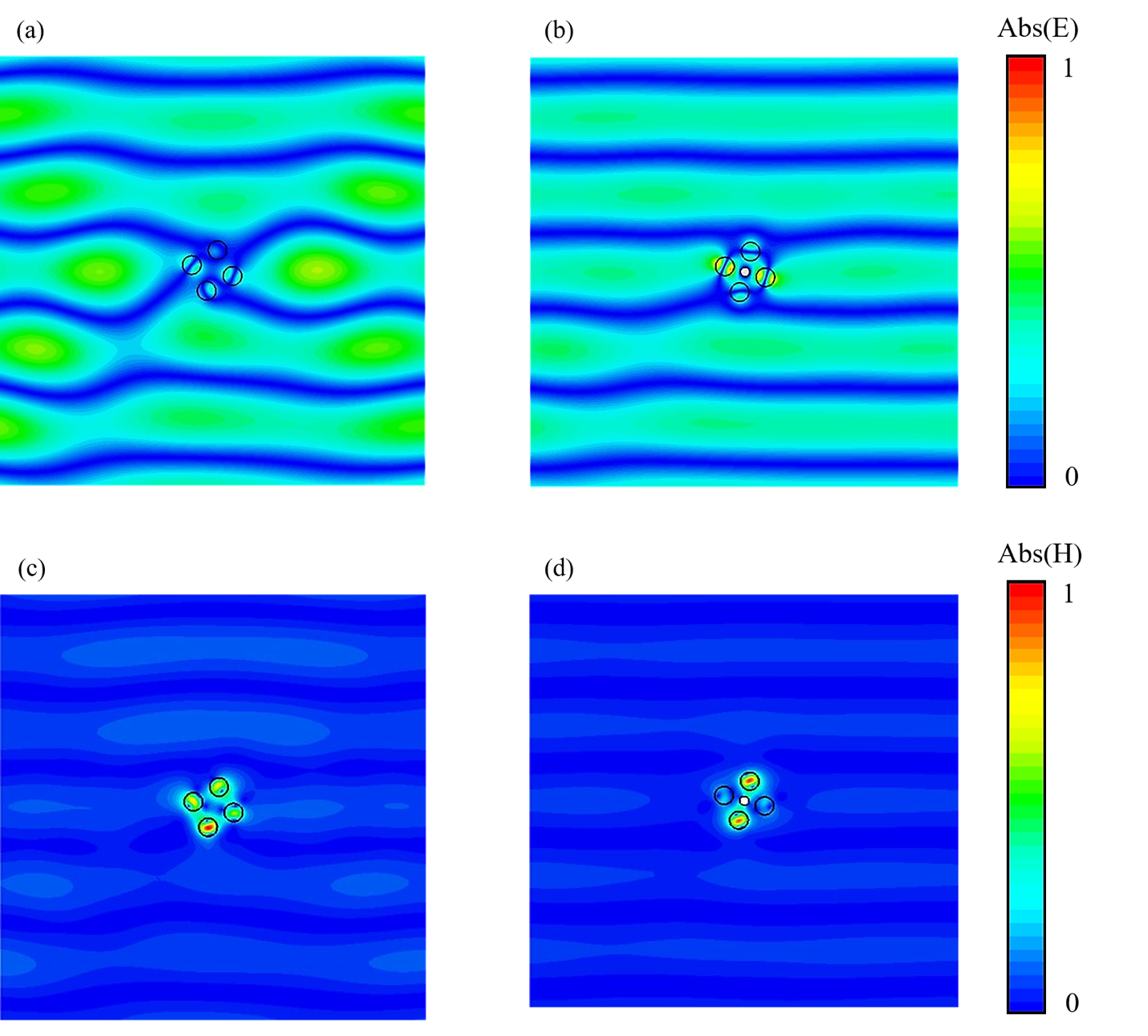


Fig. S2. Field maps of the y-component of electric field intensities and of the absolute value of magnetic field intensities of all-dielectric cover ((a) and (c)) and cloaking metamolecule ((b) and (d)) for θ=30^0^ incident wave at f=3.58 THz.

Furthermore, we provide multipolar decomposition of near field of cloaking device at resonance. Figure S3 provides multipolar decomposition up to second order of multipoles ­ **P**, **M**, **T**, **Q**e, **Q**m, scattered by all-dielectric metamolecule and cloaking device at θ=0^0^ (S3 (a) and (b)) and θ=30^0^ (S3 (c) and (d)) angles of incident wave at minimum scattering value regarding to RCS pattern (see Figure 2).

We demonstrate on Figures S3 (a) and (b) multipolar decomposition of all-dielectric cluster and cloaking device at θ=0^o^ angle of incidence. Obviously, there is anapole mode excitation at resonant frequency f=3.58 THz. Thus, the electric dipolar **P** moment dominates at low frequencies and falls dramatically from 3.55 THz. As for toroidal dipole **T**, one can see gradual increase from 3.16 THz and stay dominant from 3.58 THz to 3.63 THz. Magnetic dipole **M** and electric quadrupole **Qe** moments maintain concurring and 100 times lower than electric and toroidal dipoles. They grow rapidly from 3.55 THz up to 3.75 THz then become dominant. Magnetic quadrupole **Qm** stays growing in the whole frequency range. Such behaviour of higher order multipoles corresponds to real anapole cloaking.

Similar situation can be observed at θ=30^o^ angle of incidence. The anapole mode arrises on the frequency f=3.58 THz (Fig. S3 (d)) manifesting as RCS minimum on Fig. 2b. Moreover, other multipoles much lower at these frequencies.


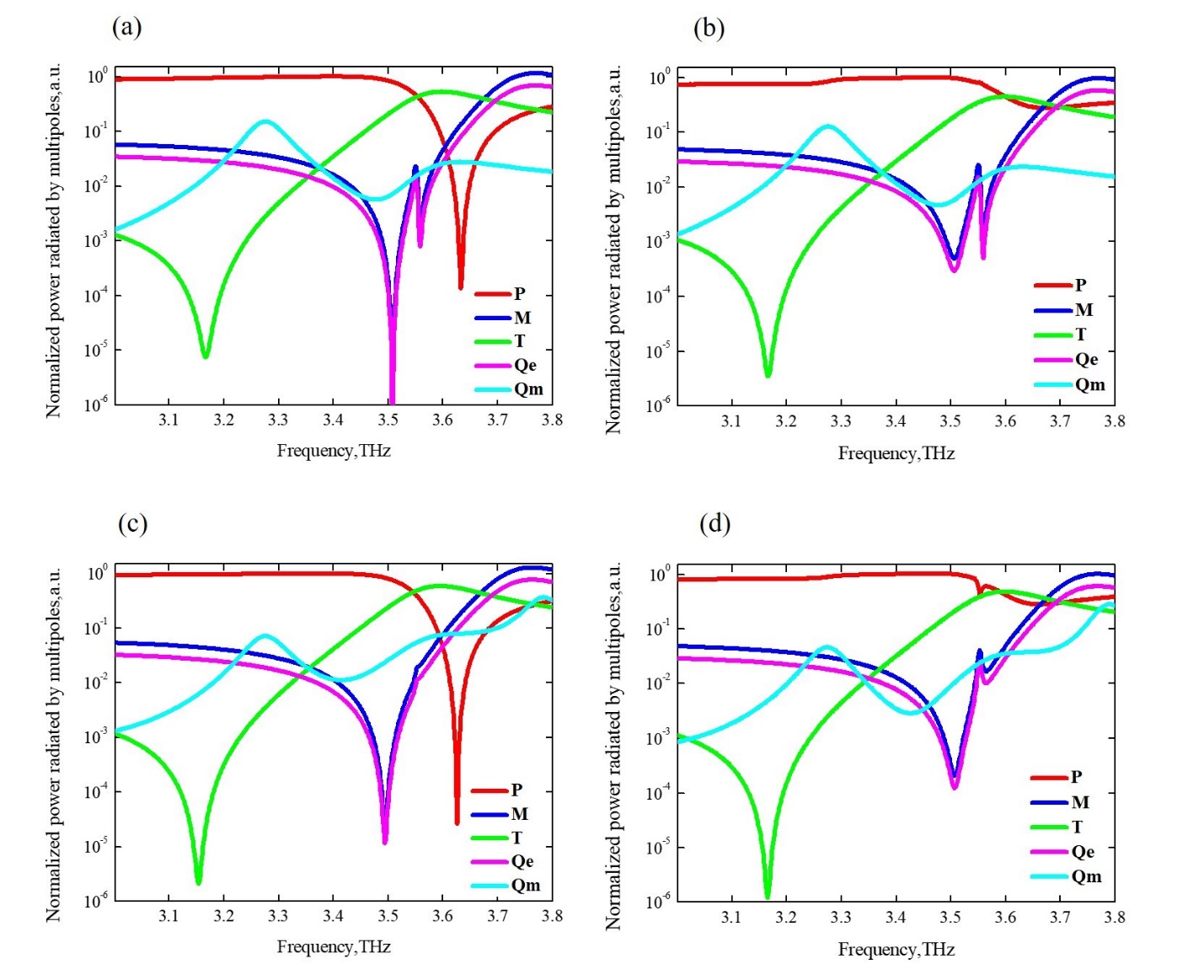


Fig. S3. Normalized power of near-field distribution of all-dielectric cover (a) and cloaking metamolecule (b) up to second order multipoles for θ=0^o^ and of all-dielectric cover (c) and cloaking metamolecule (d) for θ=30^o^ incident wave direction.

We also provide RCS calculation for single PEC scatterer (black curve), all-dielectric cluster (red curve) as well as cloaking device (blue curve) in case of perpendicular polarized incident wave at θ=45^0^ incidence angle. One can see that perpendicular polarized incident wave does not introduce strong scattering suppression at considered frequency range at all and does not represent any interest.


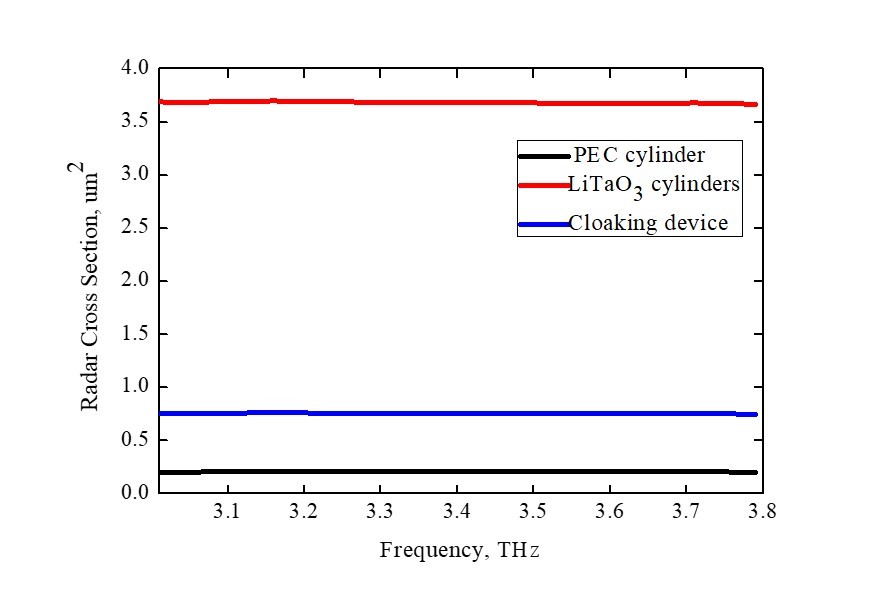


Fig. S4. Radar cross section (RCS) for “stealthiness” (black curve), all-dielectric cover (red curve) and cloaking metamolecule (blue curve) for perpendicular polarization of incident wave at θ=45^o^ incidence angle.
